# Phase I, first-in-human trial of programmed cell death receptor-1 (PD-1) inhibitor, JTX-4014, in adult patients with advanced, refractory, solid tumors

**DOI:** 10.1007/s00262-020-02730-5

**Published:** 2020-09-28

**Authors:** Kyriakos P. Papadopoulos, Nehal Lakhani, Gerald S. Falchook, Gosia Riley, Johan Baeck, Karen S. Brown, Gilad Gordon, Lidya Le, Judy S. Wang

**Affiliations:** 1 South Texas Accelerated Research Therapeutics (START), 4383 Medical Drive, Suite 4021, San Antonio, TX 78229 USA; 2 START Midwest, Grand Rapids, MI USA; 3 Sarah Cannon Research Institute at HealthONE, Denver, CO USA; 4 Jounce Therapeutics, Inc., Cambridge, MA USA; 5 Florida Cancer Specialists/Sarah Cannon Research Institute, Sarasota, FL USA

**Keywords:** Immunotherapy, Programmed cell death 1 receptor, Investigational therapies, Tumor biomarkers, Salivary gland tumors

## Abstract

**Background:**

Inhibition of programmed cell death receptor protein-1 (PD-1) has proven to be a highly effective strategy for immunotherapy of cancer. Approvals of both PD-1 and PD-L1 inhibitors [PD-(L)1i] in multiple tumor types are evidence of the durable benefits they provide to patients with cancer. In this first-in-human trial, we assessed the safety and tolerability of JTX-4014, a fully human antibody targeting PD-1.

**Methods:**

JTX-4014 was administered to 18 patients with multiple solid tumor types who had not previously received a PD-(L)1i. The primary objectives were to evaluate the safety and tolerability of JTX-4014 and determine the maximum tolerated dose (MTD) and recommended phase II dose (RP2D). Secondary objectives included evaluation of the pharmacokinetics (PK) of JTX-4014, anti-drug antibodies (ADA) against JTX-4014, and clinical activity**.**

**Results:**

JTX-4014 was well tolerated and no new safety signals were identified as compared with other PD-1is. The MTD was not reached and the RP2D was selected, based on PK modelling and supportive safety data, to be 500 mg every 3 weeks or 1000 mg every 6 weeks. Clinical activity, based on RECIST v1.1 criteria, demonstrated an overall response rate of 16.7% (*n* = 3) with one complete and two partial responses and a disease control rate of 44.4% (*n* = 8). The responses occurred at different doses in patients with PD-L1 positive tumors and in tumor types that are not typically PD-1i responsive.

**Conclusions:**

Further development of JTX-4014 is warranted as a monotherapy or in combination with other innovative cancer therapies.

**Trial registration number:**

NCT03790488, December 31 2018.

## Introduction

The approvals of PD-1 inhibitors (PD-1is), including pembrolizumab and nivolumab, have caused a paradigm shift in immuno-oncology therapeutics, providing durable remissions for many patients with cancer [1–4]. Patients who achieve objective responses can maintain durable responses for years, demonstrating the potential of the immune system to eradicate or prevent recurrence of cancer [1, 5, 6]. However, response rates to PD-1i monotherapy remain low, prompting investigation of multiple combination therapies across a wide variety of cancers [1, 7–11]. Pembrolizumab and nivolumab dominate the approved immunotherapy treatment landscape, but more PD-1is are needed to support development of innovative combination therapies.

JTX-4014 is a fully human, investigational, anti-PD-1 monoclonal antibody consisting of two identical, hinge-stabilized immunoglobulin gamma 4 heavy chains, and two identical kappa light chains. JTX-4014 specifically binds to PD-1 and augments antitumor activity by blocking the interaction between PD-1 and its ligands, PD-L1 and PD-L2. JTX-4014 is being developed in combination with other therapies for the treatment of cancer in which inhibition of PD-1 may be of benefit. This phase I first-in-human (FIH) trial was designed to evaluate the safety and tolerability of JTX-4014, along with its maximum tolerated dose (MTD) and recommended phase II dose (RP2D), to provide the foundation for future clinical development.

## Materials and methods

### Study patients

Patients were eligible for inclusion if they were ≥ 18 years, had a histologically or cytologically confirmed solid tumor that was recurrent, metastatic, or refractory to at least one prior line of therapy and had no further standard treatment options. Eligible patients were not on any concurrent anticancer treatment, had received no prior anti-PD-1 or anti-PD-L1 therapy, had an Eastern Cooperative Oncology Group (ECOG) performance status of 0 or 1, had no history of immune mediated conditions including pneumonitis, were not on active systemic corticosteroid usage > 10 mg/day, and had adequate renal, hepatic, and bone marrow function. Women who were pregnant or lactating were excluded. Of note, positive PD-L1 expression in tumor tissue was not an inclusion criterion.

### Study drug

JTX-4014 was manufactured for Jounce Therapeutics, Inc. by a contract manufacturer and was administered as a 60-min intravenous infusion either once every 3 weeks (Q3W) or once every 6 weeks (Q6W), depending on the cohort.

### Trial design

This phase I, open-label, dose-escalation, FIH study (NCT03790488) was designed to evaluate the safety, tolerability, and pharmacokinetics (PK) of JTX 4014 when administered as a single agent to adult patients with advanced, refractory, solid tumors. The trial was approved by the institutional review boards at each of the four participating sites. The trial design followed a traditional 3 + 3 dose-escalation design. Dose escalation could only proceed after review of safety and tolerability and dose limiting toxicities (DLT, pre-defined and occurring in the first cycle) with the investigators and sponsor after at least three patients in each cohort completed a 21-day DLT period. Dosing groups were as follows: Cohort 1: 80 mg Q3W; Cohort 2: 240 mg Q3W; Cohort 3a: 800 mg Q3W; Cohort 3b: 800 mg Q6W; Cohort 3c: 400 mg Q3W; and Cohort 4: 1200 mg Q3W. Once a RP2D was determined, patients receiving and tolerating a different dose were eligible to increase their dose to the RP2D, at the discretion of the investigator.

### Study objectives

The primary objectives were to evaluate the safety and tolerability of JTX-4014 and determine the MTD and RP2D. The secondary objectives were to evaluate the PK of JTX-4014 and anti-drug antibodies (ADA) against JTX-4014. Other secondary objectives included clinical activity of JTX-4014 and duration of response.

### Pharmacokinetics

Blood samples for PK were collected pre-dose and at 1 (end of infusion), 24, 48, 168, 336, and 504 hours (h) post-dose for cycle 1 and cycle 3 and pre-dose, and 1 h post-dose (end of infusion) for cycles 2 and 4 through 9. JTX-4014 concentrations were determined by a validated enzyme-linked immunosorbent assay-based assay. PK parameters following first dose were calculated by non-compartmental analysis using Phoenix WinNonlin™ Version 8.2 (Certara USA, Inc).

A population pharmacokinetic (PopPK) analysis was performed using a non-linear mixed effects modelling approach and data from 18 patients treated with JTX-4014. Model selection was based on the objective function value (OVF), goodness of fit plots, and scientific plausibility. Body weight was investigated as a covariate of clearance and volume of distribution but was not significant (i.e., it did not result in a reduction in the OFV of 6.63 or more [*P* < 0.01, degree of freedom = 1]). Reliability of the model was evaluated based on diagnostic plots, visual predictive checks, and assessment of parameter uncertainty. Simulations were performed to characterize exposure profiles for various potential phase II dosing regimens. Interindividual variability random effects on CL, V1 and V2 were included as well as a covariance of V1 and V2. A proportional residual error model was employed.

### Statistical analysis

#### Safety analyses

Adverse events (AEs) were classified according to the most recent version of the Medical Dictionary for Regulatory Activities (MeDRA) and were graded for severity per common terminology criteria for adverse events (CTCAE) 5.0. Incidence [N (%)] of treatment-emergent AEs (TEAEs) are presented by System Organ Class preferred term and CTCAE toxicity grade. PK and immunogenicity data were summarized by descriptive statistics.

#### Efficacy analyses

Antitumor activity was assessed both clinically and using imaging (computed tomography scan), unless contraindicated, including optional brain magnetic resonance imaging at screening, and every 9 weeks (± 7 days) after initial dose. Radiological response was assessed by the investigator using RECIST v1.1.

## Results

### Patient demographics

From December 2018 to May 2019, 18 patients were enrolled in the study, and all were included in the final safety and efficacy analyses. As of the data cut-off date, April 15, 2020, 16 patients had discontinued and 2 patients were ongoing. The patients included ten males and eight females, with an average age of 66 years (Table 1). The patients were heavily pre-treated with a median of 3 (1–13) prior regimens. The most common tumor types enrolled were ovarian (*n* = 4), sarcoma (*n* = 3), mesothelioma (*n* = 2), prostate, (*n* = 2) and salivary gland (*n* = 2).

**Table 1:** Patient demographics at baseline

	Cohort 1 (80 mg Q3W) (*n* = 3)	Cohort 2 (240 mg Q3W) (*n* = 3)	Cohort 3c (400 mg Q3W) (*n* = 3)	Cohort 3a (800 mg Q3W) (*n* = 3)	Cohort 3b (800 mg Q6W) (*n* = 3)	Cohort 4 (1200 mg Q3W) (*n* = 3)		Total (*N* = 18)
Sex, *n* (%)								
Male	2 (66.7)	2 (66.7)	2 (66.7)	1 (33.3)	1 (33.3)	2 (66.7)		10 (55.6)
Female	1 (33.3)	1 (33.3)	1 (33.3)	2 (66.7)	2 (66.7)	1 (33.3)		8 (44.4)
Age (years)								
Mean (SD)	69.0 (6.00)	60.0 (7.94)	73.0 (6.24)	65.3 (24.42)	69.0 (8.72)	61.7 (8.96)		66.3 (11.24)
Ethnicity, *n* (%)								
Hispanic or Latino	1 (33.3)	1 (33.3)	0	1 (33.3)	1 (33.3)	0		4 (22.2)
Not Hispanic or Latino	2 (66.7)	2 (66.7)	3 (100.0)	2 (66.7)	2 (66.7)	3 (100.0)		14 (77.8)
Race, *n* (%)								
Asian	0	0	1 (33.3)	0	0		0	1 (5.6)
White	3 (100.0)	3 (100.0)	2 (66.7)	3 (100.0)	3 (100.0)		3 (100.0)	17 (94.4)
Prior therapies								
Median (min, max)	2.0 (1.0, 9.0)	4.0 (2.0, 13.0)	4.0 (1.0, 5.0)	3.0 (1.0, 6.0)	3.0 (1.0, 3.0)		3.0 (1.0, 6.0)	3.0 (1.0, 13.0)
ECOG, *n* (%)								
0	1 (33.3)	1 (33.3)	1 (33.3)	1 (33.3)	2 (66.7)		0	6 (33.3)
1	2 (66.7)	2 (66.7)	2 (66.7)	2 (66.7)	1 (33.3)		3 (100.0)	12 (66.7)
Tumor type, *n* (%)								
Ovary	1 (33.3)	0	1 (33.3)	0	1 (33.3)		1 (33.3)	4 (22.2)
Sarcoma	0	1 (33.3)	0	1 (33.3)	0		1 (33.3)	3 (16.7)
Mesothelioma	0	1 (33.3)	0	0	1 (33.3)		0	2 (11.1)
Prostate	1 (33.3)	0	0	0	0		1 (33.3)	2 (11.1)
Salivary gland	0	0	1 (33.3)	0	1 (33.3)		0	2 (11.1)
Other^a^	1 (33.3)	1 (33.3)	1 (33.3)	2 (66.7)	0		0	5 (27.8)

*SD* standard deviation

^a^Others included: breast, esophageal, gastric, neuroendocrine carcinoma, and pelvic (1 each)

### Safety

JTX-4014 was well tolerated, resulted in no treatment-related deaths or DLTs and had an acceptable safety profile. The MTD was not identified inclusive of the maximum administered dose of 1200 mg Q3W. Sixteen patients experienced 2 or more TEAEs, as shown in Table 2. The most common TEAEs were fatigue (50.0%, *n* = 9), aspartate aminotransferase increase (22.2%, *n* = 4) and dizziness (22.2%, *n* = 4). Of the four patients with dizziness, two events were unrelated to JTX4014 and two were attributed to JTX4014, including in one patient an infusion-related reaction with dizziness.

**Table 2:** Summary of Grade 1/2 TEAEs that occurred in two or more patients and all Grade 3/4 TEAEs

Preferred Term	Grade 1/2 (*n* = 18)	Grade 3/4 (*n* = 18)^b^	Any grade (*n* = 18)
Patients with at least 1 TEAE, n (%)	16 (88.9)	7 (38.9)	16 (88.9)
Fatigue	9 (50.0)	0	9 (50.0)
Dizziness	4 (22.2)	0	4 (22.2)
Aspartate aminotransferase increase	4 (22.2)	0	4 (22.2)
Alanine aminotransferase increase	3 (16.7)	0	3 (16.7)
Anemia	2 (11.1)	1 (5.6)	3 (16.7)
Tumor pain	2 (11.1)	1 (5.6)	3 (16.7)
Edema peripheral	2 (11.1)	0	2 (11.1)
Pyrexia	2 (11.1)	0	2 (11.1)
Headache	2 (11.1)	0	2 (11.1)
Cough	2 (11.1)	0	2 (11.1)
Decreased appetite	2 (11.1)	0	2 (11.1)
Dehydration	2 (11.1)	0	2 (11.1)
Nausea	2 (11.1)	0	2 (11.1)
Pruritus	2 (11.1)	0	2 (11.1)
Back pain	2 (11.1)	0	2 (11.1)
Blood creatinine increased	1 (5.6)	1 (5.6)	2 (11.1)
Blood alkaline phosphate increased^a^	0	1 (5.6)	1 (5.6)
Chronic obstructive pulmonary disease	0	1 (5.6)	1 (5.6)
Deafness unilateral	0	1 (5.6)	1 (5.6)
Lymphocyte count decreased	0	1 (5.6)	1 (5.6)
Metastases to central nervous system	0	1 (5.6)	1 (5.6)
Pneumonitis^a^	0	1 (5.6)	1 (5.6)
Pneumothorax	0	1 (5.6)	1 (5.6)
Rash maculo-papular^a^	0	1 (5.6)	1 (5.6)
Urinary tract obstruction	0	1 (5.6)	1 (5.6)
Venous thrombosis	0	1 (5.6)	1 (5.6)

*TEAE* treatment-emergent adverse events

^a^Grade 3 events attributed by the investigator as related to the study drug

^b^All Grade 3/4 events are Grade 3 AEs. There were no Grade 4 AEs reported

Seven patients (38.9%) experienced a total of 13 Grade ≥ 3 TEAEs (Table 2). Six of these 13 events occurred in 2 patients at the highest dose of 1200 mg Q3W. All Grade ≥ 3 TEAEs occurred at a relatively low frequency, with no single Grade 3 TEAE being reported in more than one patient. Only three Grade ≥ 3 AEs were attributed to the study drug, namely pneumonitis at 1200 mg Q3W, a maculopapular rash at 800 mg Q3W and an isolated increase in blood alkaline phosphatase at 240 mg Q3W. Pneumonitis, the only drug-related serious AE, occurred after the second dose in a patient with recurrent pleural effusions. This patient presented with bilateral lung opacities and was treated with corticosteroids, with clinical resolution of the event after 3 days. Four patients (22.2%) discontinued treatment due to an AE or serious AE, one patient each at 80 mg Q3W and 800 mg Q3W, and two at 1200 mg Q3W.

### Pharmacokinetics

**Fig. 1:**
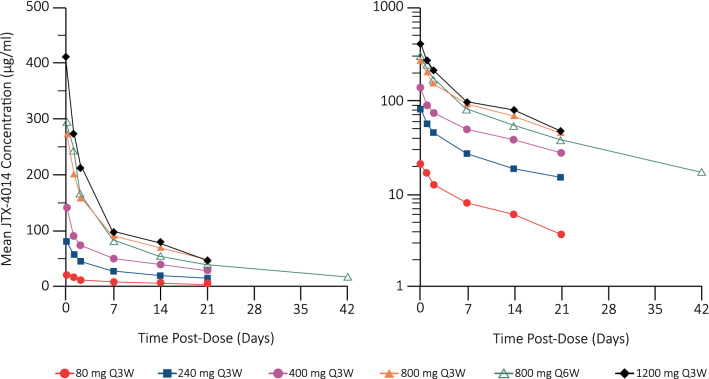
JTX-4014 cycle 1 pharmacokinetics in patients with solid tumors. Mean cycle 1 JTX-4014 concentrations are plotted on a linear (left) and logarithmic (right) scale versus time post-dose

JTX-4014 exhibited linear PK between 80 and 1,200 mg (Fig. 1), with dose-proportional increases in both maximum concentration (C_max_) and area under the curve. The mean terminal half-life (T_1/2_) ranged from 7 to 14 days and steady state was attained by cycle 7. The pharmacokinetic characteristics of JTX-4014 were described by a two-compartment model and linear elimination. Inclusion of body weight as a covariate did not result in a statistically significant improvement of the model. The total volume of distribution was estimated to be approximately 6 L. The terminal half-life by PopPK modeling was predicted to be approximately 17 days. No ADAs were detected in any patients as of the data cut-off.

Pharmacokinetic data from 18 patients, comprising 221 JTX-4014 concentrations, were used for the PopPK analysis. As the PK of JTX-4014 was similar to that reported for approved PD-1is (pembrolizumab and nivolumab), simulations were conducted to identify RP2D regimens that would achieve median trough concentrations at steady state (C_trough,ss_) for JTX-4014, comparable to or greater than the pembrolizumab mean/median C_trough,ss_ and the nivolumab geometric mean C_trough,ss_. Based on these simulations, two RP2D regimens were identified: 500 mg Q3W (simulated median [95% CI] C_trough,ss_ of 47.46 [22.12, 96.51] µg/mL, or 1000 mg Q6W (simulated median [95% CI] C_trough,ss_ of 29.70 [8.77, 71.24] µg/mL).

### Clinical activity

**Fig. 2:**
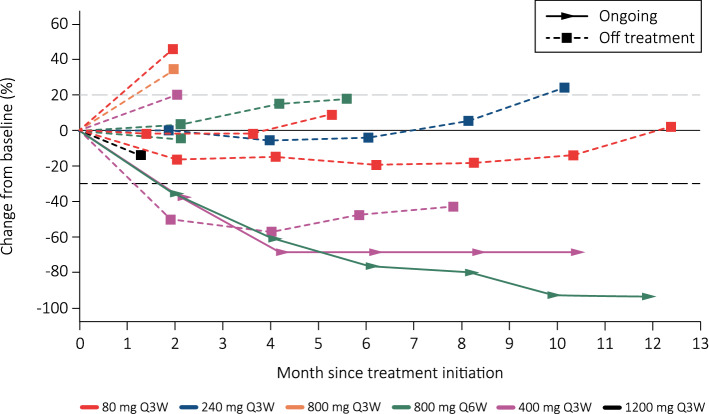
Percent change from baseline in sum of tumor diameter. This figure excludes four patients who did not have a post-baseline scan and two patients who did not have measurable lesions

**Fig. 3:**
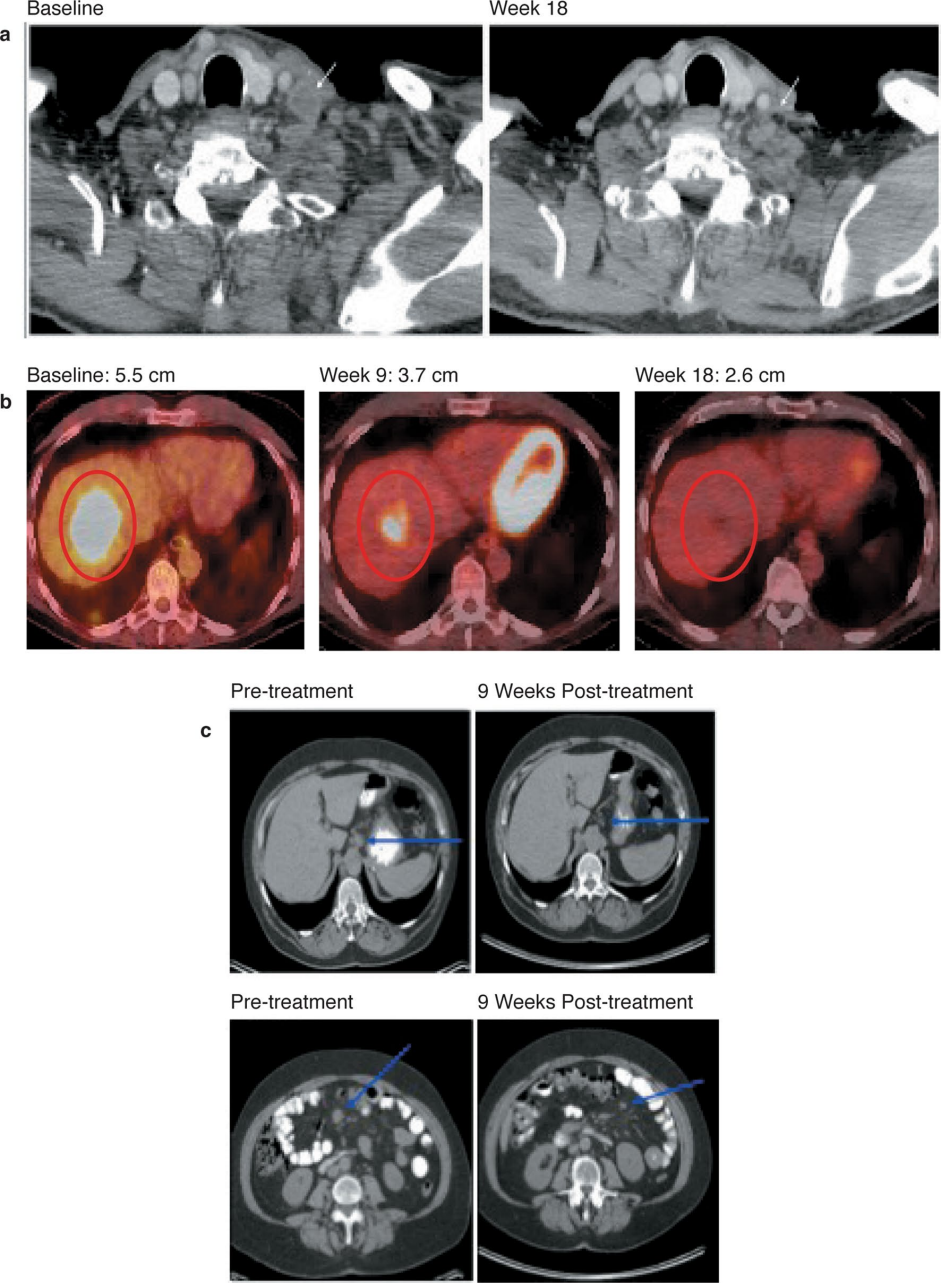
**a** Mucoepidermoid carcinoma of the parotid (400 mg Q3W)—confirmed complete response. **b** Carcinoma ex-pleomorphic adenoma (800 mg Q6W)—confirmed partial response. **c** Ovarian carcinoma (400 mg Q3W)—confirmed partial response

JTX-4014 elicited meaningful responses in this heavily pre-treated population (Table 3 and Fig. 2). Based on RECIST v1.1 criteria by investigator assessment, three patients (16.6%) had confirmed responses, 1 complete response (CR) and 2 partial responses (PRs) (Fig. 3). In addition, five patients (27.8%) had a best response of stable disease for an overall disease control rate of 8/18 (44.4%). Six patients (33.3%) progressed at the time of their first radiological evaluation. The remaining four patients (22.2%) discontinued prior to the first radiological evaluation, two due to unrelated AEs, one due to investigator decision and one due to clinical progression. The overall median number of cycles of JTX-4014 that was administered was 3.5 (range 1–18).

**Table 3:** Best overall response and overall response rate – safety population

	Cohort 1 (80 mg Q3W) (*n* = 3)	Cohort 2 (240 mg Q3W) (*n* = 3)	Cohort 3c (400 mg Q3W) (*n* = 3)	Cohort 3a (800 mg Q3W) (*n* = 3)	Cohort 3b (800 mg Q6W) (*n* = 3)	Cohort 4 (1,200 mg Q3W) (*n* = 3)	Total (*N* = 18)
Best overall response, *n* (%)	0	0	0	0	0	0	0
Complete response	0	0	1 (33.3)	0	0	0	1 (5.6)
Partial response	0	0	1 (33.3)	0	1 (33.3)	0	2 (11.1)
Stable disease	2 (66.7)	(2 (66.7)	0	0	1 (33.3)	0	5 (27.8)
Progressive disease	1 (33.3)	0	1 (33.3)	2 (66.7)	1 (33.3)	1 (33.3)	6 (33.3)
Early termination	0	1 (33.3)	0	1 (33.3)	0	2 (66.7)	4 (22.2)
Overall response rate, *n* (%)	0	0	2 (66.7)	0	1 (33.3)	0	3 (16.7)
95% confidence interval	(0.00 to 70.76)	(0.00, 70.76)	(89.43 to 99.16)	(0.00 to 70.76)	(0.84 to 90.57)	(0.00 to 70.76)	(3.58 to 41.42)
Disease control rate, *n* (%)	2 (66.7)	2 (66.7)	2 (66.7)	0	2 (66.7)	0	8 (44.4)
95% confidence interval	(9.43 to 99.16)	(9.43 to 99.16)	(9.43 to 99.16)	(0.00 to 70.76)	(9.43 to 99.16)	(0.00 to 70.76)	(21.53 to 69.24)

The three patients who exhibited either a complete or partial response all had evidence of PD-L1 expression on their tumors. Of note, among the remaining 15 patients, 1 patient had PD-L1 staining of 0% and the rest had no PD-L1 staining available. The patient who had a CR was an 80-year-old male with a mucoepidermoid carcinoma of the parotid that stained 60% for PD-L1, who was previously treated with surgery, radiation, and a single previous regimen containing carboplatin and cetuximab (Fig. 3a). His response on 400 mg Q3W was still ongoing at 338 days as of the data cut-off. One of the patients with a PR was a 63-year-old male with carcinoma ex-pleomorphic adenoma that stained 100% for PD-L1, who was previously treated with surgery, radiation, and three previous regimens, which, in total, consisted of pertuzumab, trastuzumab, leuprolide, carboplatin, paclitaxel, and bicalutamide (Fig. 3b). His response on 800 mg Q6W was still ongoing at 386 days as of the data cut-off. The second patient with a PR was a 68-year-old woman with ovarian cancer that stained 5% for PD-L1, who was previously treated with surgery and four previous regimens, which, in total, consisted of carboplatin, paclitaxel, gemcitabine, bevacizumab, docetaxel and an investigational agent (Fig. 3c). Time to progression on 400 mg Q3W was 232 days.

Of the five patients who had a best response of stable disease, the mean duration of the stable disease was 230 days (range 127–378 days) with a median of 170 days. One of the patients was noted to have progressive disease (PD) based on radiological findings at day 63; however, the patient was felt to have clinical benefit and, per protocol, was maintained on the study and remained stable for a total of 282 days. Per RECIST 1.1, the patient was considered to have PD even though she stayed on trial beyond the first radiological examination.

## Discussion

This FIH trial of a new PD-1i, JTX 4014, demonstrated that the drug was well tolerated, has an acceptable safety profile and is clinically active. There were no deaths, no DLTs and the only related serious AE was pneumonitis, which occurred after the second dose (after the DLT period) in the highest cohort of 1200 mg Q3W. Overall, the safety results were comparable to the reported safety results from other PD-1i with no new safety signals and no evidence of ADAs [12–14]. There were three radiologically confirmed responses (one CR and two PRs) at 400 mg Q3W and 800 mg Q6W, who all had tumors that stained positive for PD-L1. Based on these safety data and PK modelling, the RP2D was determined to be either 500 mg Q3W or 1000 mg Q6W.

A noteworthy finding of our study is the clinical benefit obtained following JTX 4014 treatment in one patient each, with carcinoma ex-pleomorphic adenoma and mucoepidermoid cancer. Both of these rare salivary gland tumors were PD-L1 > 50% and the patients have durable and ongoing responses. Among salivary tumors, high PD-L1 expression is associated with high-grade tumors and possibly worse clinical outcomes [15–17]. PD-L1 expression in carcinoma ex-pleomorphic adenoma and mucoepidermoid tumors occurs in 10–75% and 9–57% of cases, respectively [15–17]. These tumors are not typically responsive to PD-1 inhibition. Of three PD-L1-positive mucoepidermoid cancers enrolled in the KEYNOTE 028 trial and treated with pembrolizumab, none demonstrated reduction in tumor size [18]. Similarly, none of three mucoepidermoid or two carcinoma ex-pleomorphic adenoma patients (of unknown PD-L1 status) had objective responses with pembrolizumab and vorinostat [19].

An additional noteworthy observation in this phase I trial was that, in this population of heavily pre-treated patients with multiple tumor types, the three responders to JTX-4014 had tumors that expressed PD-L1 in tumor types that are not typically PD-Li responsive. This supports the need for predictive biomarkers to select patients from less typically PD-1i responsive tumor types who will respond to a PD-1i.

Results of this trial indicate that JTX-4014 may offer clinical benefit to cancer patients as a monotherapy. Furthermore, JTX-4014 may provide an option for combination therapy with other novel agents.

In summary, these data demonstrate that JTX-4014 is well tolerated, with a similar safety profile to that seen with other PD-1is [12–14]. In addition, JTX-4014 was shown to be clinically active in a heavily pre-treated patient population with multiple tumor types. This trial identified two different dosing schedules for the RP2D, which should enable further development of JTX-4014 in combination with innovative cancer therapies. Further study is warranted with innovative combinations and potential predictive biomarkers.

## Abbreviations

ADA: Anti-drug antibodies
AE: Adverse event
CL: Clearance
CR: Complete response
CTCAE: Common terminology criteria for adverse events
DLT: Dose limiting toxicities
ECOG: Eastern Cooperative Oncology Group
MeDRA: Medical Dictionary for Regulatory Activities
MTD: Maximum tolerated dose
OVF: Objective function value
PD-1: Programmed cell death receptor protein-1
PD1i: PD-1 inhibitor
PK: Pharmacokinetics
PR: Partial response
Q3W: Once every 3 weeks
RP2D: Recommended phase II dose
TEAE: Treatment-emergent adverse event
